# Theoretical Studies on the Isomerization Kinetics of Low-Lying Isomers of the SiC_4_H_2_ System

**DOI:** 10.1021/acs.jpca.3c05658

**Published:** 2023-12-20

**Authors:** Nisha Job, Vijayanand Chandrasekaran, Venkatesan S. Thimmakondu, Krishnan Thirumoorthy

**Affiliations:** † Department of Chemistry, School of Advanced Sciences, Vellore Institute of Technology, Vellore, Tamil Nadu 632 014, India; ‡ Department of Chemistry and Biochemistry, San Diego State University, San Diego, California 92182-1030, United States

## Abstract

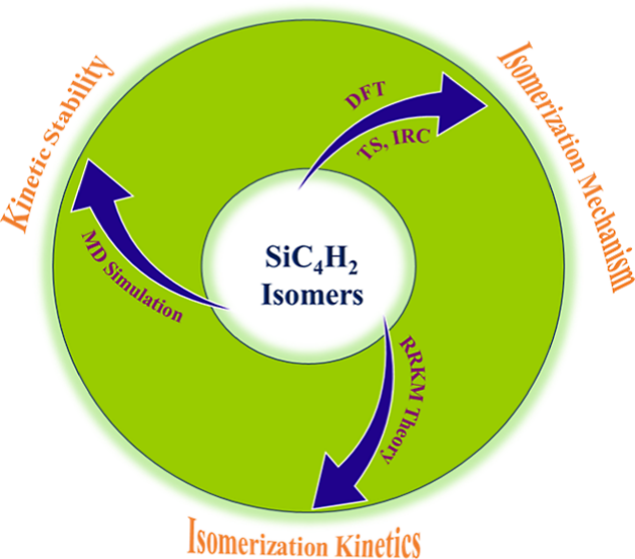

The low-lying isomers of SiC_4_H_2_ are investigated to understand the kinetics of isomerization pathways using density functional theory. In our earlier work, we studied the various possible isomers (*J. Phys. Chem. A*, **2020**, 124, 987–1002) and the chemical bonding of low-lying isomers of SiC_4_H_2_ (*J. Phys. Chem. A*, **2022**, 126, 9366–9374). Among them, four isomers, 1-ethynyl-3-silacycloprop-1-en-3-ylidene (**1**), 3-silapent-1,4-diyn-3-ylidene (**2**), 1-silapent-1,2,3,4-tetraen-1-ylidene (**4**), and 1-silapent-2,4-diyn-1-ylidene (**5**) have already been identified in the laboratory. The previously known theoretical isomer 2-methylene-1-silabicyclo[1.1.0]but-1(3)-en-4-ylidene (**3**) and the newly identified unknown isomer through the present kinetic studies 5-silabicyclo[2.1.0]pent-1(4),2-dien-5-ylidene (**N6**) remain elusive in the laboratory to date. The isomerization pathways of the low-lying isomers of SiC_4_H_2_ are predicted through the transition state structures. Intrinsic reaction coordinate analysis identifies the minimum energy reaction pathways connecting the transition state from one isomer to another of the investigated system. The present kinetic data reveal the isomerization of global minimum energy isomer **1** to thermodynamically stable low-lying isomers, **2** and **5**. Interestingly, isomer **3** interconverts to the experimentally known low-energy isomer **4**, the second most thermodynamically stable isomer among them. The thermodynamic and kinetic parameters of the low-lying isomers of SiC_4_H_2_ are also documented in this work. The rate coefficient and equilibrium constant for isomerization reactions are calculated using the Rice–Ramsperger–Kassel–Marcus theory. The equilibrium constant delineates the difficulties in forming **N6** and **3** through the isomerization pathways. Furthermore, ab initio molecular dynamics studies dictate the stability of low-lying isomers of SiC_4_H_2_ within the time scale of the simulation.

## 1 Introduction

In recent years, group 14 elements, such as carbon and silicon, have been found to be essential in advancing astrophysics and astrochemistry due to their proven existence in the interstellar medium (ISM) and circumstellar envelopes (CSEs).^1−5^ Molecules containing silicon account for roughly 10% of the molecular species in space. Specifically, silicon-bearing compounds are essential for gas-orbiting star-forming regions and late-type stars.^6−9^ Out of the 300 species identified in ISM and CSEs, 15 are Si-bearing species.^10,11^ One can understand the chemical evolution of carbon-rich CSEs through astronomical observations of these molecules and their astrochemical model exploiting intricate gas phase reaction networks.^12−19^ Complex organosilicon molecules are abundant in the asymptotic giant branch (AGB) CSEs, but their formation mechanisms remain an open debate.^20−22^ The significance of organosilicon molecules as precursors in forming silicon-carbide dust grains in CSE discharge has garnered special attention. It is unknown how the chemistry of silicon and carbon around stars has led to the formation of submicron-sized granules.^23^ Stellar winds and outflows cause evolved stars to lose mass in the latter phases of their evolution. These outflows may include silicon-bearing molecules, in addition to gas and dust. In addition, shock waves provide energy, and pressure conditions lead to the formation of these molecules.^24−27^ Hydrogenated organosilicon compounds may form due to the interaction between extruding molecules and the surrounding environment. The specific pathways and mechanisms for hydrogenated organosilicon compound formation are an ongoing new frontier in research. In previous studies, related elemental compositions such as SiC_2_H_2_, SiC_3_H_2_, SiC_3_H_4,_ Si_2_CH_2_, Si_2_C_5_H_2,_ and Si_3_C_2_H_2_ are studied, and plausible synthetic routes and spectroscopic parameters were either reported experimentally or theoretically.^21−30^ Till now, more than five hundred absorption bands called diffuse interstellar bands (DIBs) have been identified. Except for C_60_ and its cation, its carriers are unknown. It is widely accepted that the carriers of diffuse interstellar bands are molecules, mostly in the gas phase. Therefore, investigating more about these molecules in astronomical observations, laboratory experiments, and theoretical predictions will shed light on the formation of these intriguing compounds. Recent laboratory studies focus on the formation mechanism of SiC_4_H_2_ molecules in different pathways, including (i) diacetylene (C_4_H_2_) and silylidyne radical (SiH) single-collision and (ii) silicon and diacetylene bimolecular reactions, which are pursued in the laboratory investigations of SiC_4_H_2_ formation.^31−39^ Since one SiC_4_ isomer is already identified in the CSE,^40^ their hydrogenated derivatives, such as SiC_4_H_2_ isomers, are considered to have potential implications in astronomical identification.

**Figure 1:**
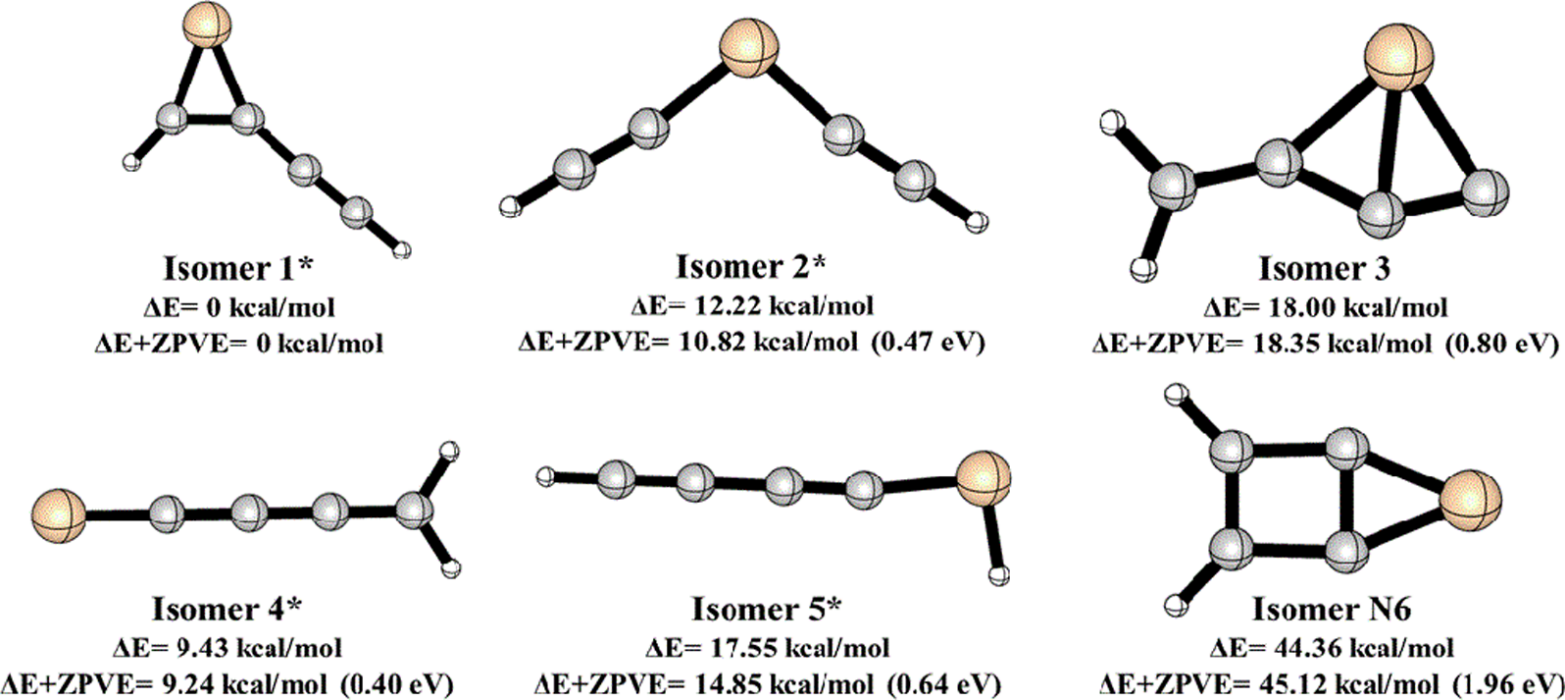
Low-lying isomers of SiC_4_H_2_ at the B3LYP/6-311++G(2d,2p) level. (The naming convention of the isomers is followed as per the previously published papers. The experimentally detected isomers are marked with an asterisk).

The present work explores the dissociation pathways of low-lying isomers of SiC_4_H_2_ as shown in Figure 1, namely, 1-ethynyl-3-silacycloprop-1-en-3-ylidene (**1**), 3-silapent-1,4-diyn-3-ylidene (**2**), 2-methylene-1-silabicyclo[1.1.0]but-1(3)-en-4-ylidene (**3**), 1-silapent-1,2,3,4-tetraen-1-ylidene (**4**), 1-silapent-2,4-diyn-1-ylidene (**5**), and 5-silabicyclo[2.1.0]pent-1(4),2-dien-5-ylidene (**N6**). Maier and co-workers^41^ had trapped **1** and **2** in the Ar matrix at 10 K by flash pyrolysis of triethynylsilane [HSi(C_2_H)_3_] and 1,1-diethynyl 2,2,2-trimethyl disilane [Me_3_Si–SiH (C_2_H)_2_]. Isomer **5** is formed by irradiating 254 nm photons (4.88 eV) on isomers **1** and **2**, causing photoisomerization.^41^ McCarthy and co-workers observed the rotational spectrum of isomer **4** in a pulsed supersonic molecular beam by Fourier transform microwave (FTMW) spectroscopy.^42^ In the same direction, thermochemistry and kinetic studies of SiC_4_H_2_ isomers on its potential energy surface (PES) are essential to understanding the possible rearrangements between various low-lying isomers and are explored in detail in the present work.

In our earlier work, the possible isomers of SiC_4_H_2_ have been reported with different levels of theoretical methods^43^ and, later, explored the chemical bonding features of SiC_4_H_2_ isomers.^44^ Out of the reported isomers, **1** is a global minimum geometry, and all other isomers of SiC_4_H_2_ lie within 50 kcal/mol, which was well documented with their structural parameters. In continuation of our previous works, the present work aims to determine the isomerization pathways of low-lying isomers of SiC_4_H_2_. The relative energies (Δ*E*), activation energies (Δ*E*^‡^), zero-point vibrational energy corrections (ZPVE), and Gibbs free energies (G) for dissociation pathways of low-lying SiC_4_H_2_ isomers are presented here, which give glimpses of their thermodynamic and kinetic stabilities. The present theoretical studies on transition state structure predictions and their minimum-energy reaction paths are performed through intrinsic reaction coordinate (IRC) analysis, which provides insights into the kinetic stability of these isomers and their isomerization pathways through various rearrangements.

## 2 Computational Methodologies

The low-lying geometries of SiC_4_H_2_ isomers considered in this work were optimized using density functional theory (DFT) at the B3LYP^45,46^/6-311++G(2d,2p)^47,48^ level of theory. We identified the transition state structures for the predicted dissociation pathways of low-lying isomers of SiC_4_H_2_ at the same level of theory. Through frequency calculations at the same level, transition state structures were confirmed with one imaginary frequency. Furthermore, the predicted dissociation pathway for each isomer was established by intrinsic reaction coordinate (IRC)^49,50^ calculations and was carried out at the same level. All the computational calculations were carried out with the Gaussian suite of programs.^51^ To investigate the kinetic stability of isomers, we executed ab initio molecular dynamics (AIMD) simulations using the atom-centered density matrix propagation (ADMP)^52^ method implemented in the Gaussian 16 program.^51^ The rate coefficients for the isomerization reaction were calculated using Rice–Ramsperger–Kassel–Marcus (RRKM) theory^53^ using the following equation
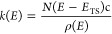
where *E*_TS_ is the energy of the transition state from the ground state of the isomers under consideration, *E* is the total energy of the isomer, *N*(*E* – *E*_TS_) is the sum of states of the transition state that would be available for the given energy *E* of the isomer, c is the velocity of light, and ρ is the density of the vibrational states. The Beyer–Swinehart (BS) algorithm is a direct count method to calculate the vibrational density of states.^54^ In this approach, the density of states was calculated by summating all of the energies of the individual harmonic oscillator for a particular energy. In this work, harmonic vibrational frequencies were used for calculating the level densities for each isomer, and the bin size for the calculation was kept as 1 cm^–1^.

## 3 Results and Discussion

### 3.1 Isomerization Pathways

**Figure 2:**
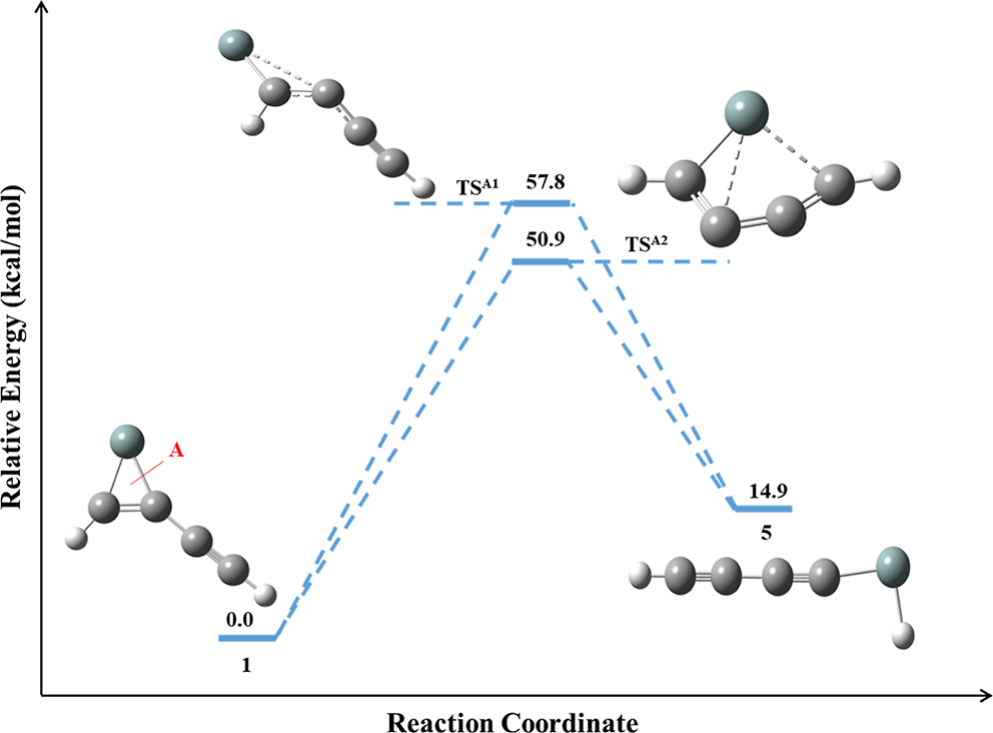
Reaction energy profiles for the isomerization of **1** to **5**. The ZPVE-corrected relative energy is in kcal/mol.

**Figure 3:**
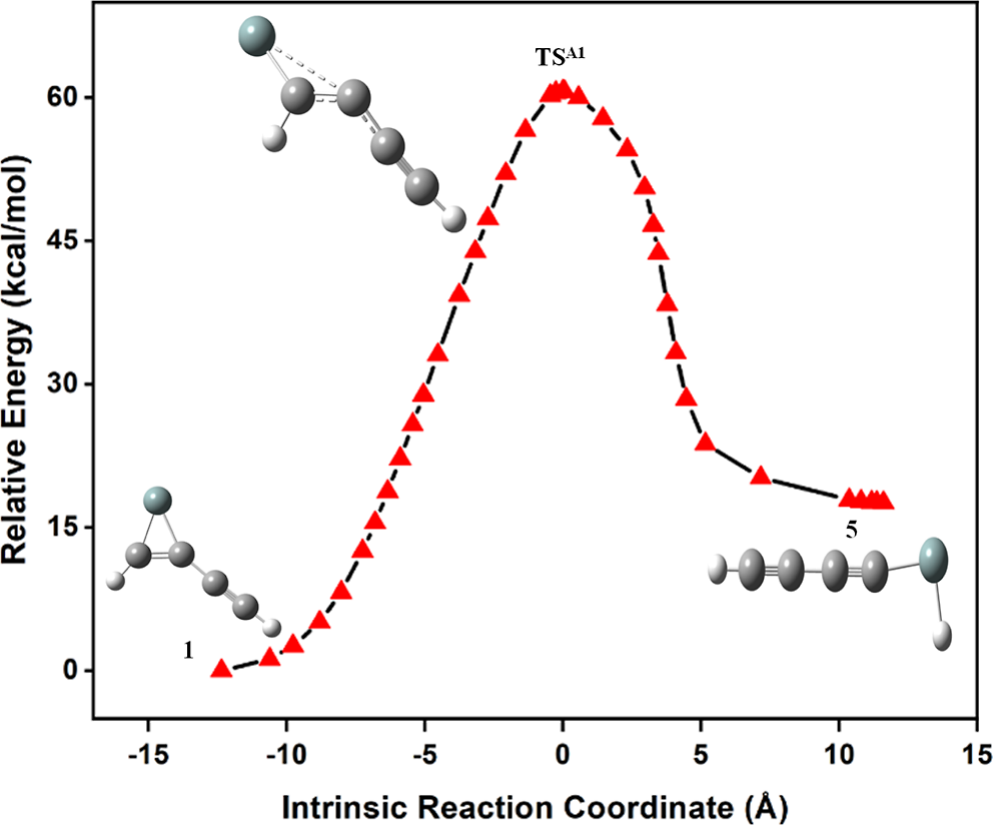
IRC for the isomerization pathway of **1** to **5**.

The reaction energy profile for the isomerization of **1** to **5** is shown in Figure 2. The dissociation pathway, type-A in **1**, leads to **5** with an activation barrier of 57.8 kcal/mol. The IRC calculation for the isomerization pathway of **1** to **5** is shown in Figure 3, which reveals that the identified transition state structure (**TS**^**A1**^) connects both **1** and **5** through the minimum energy reaction path. The formation of **5** happens by breaking the Si–C single bond in **1**. The optimized geometrical parameters for the isomerization pathway of **1** to **5** and its corresponding **TS**^**A1**^ are compared in Table S1. From the reaction energy profile, it is clear that the formation of **5** from **1** is endothermic. Furthermore, the identified transition state, **TS**^**A2**^, also connects the global minimum geometry **1** and **5**. The optimized geometrical parameters for the isomerization pathway of **1** to **5** and its corresponding **TS**^**A2**^ are compared in Table S2. The computed results of the reaction energy profile predict that the mechanism behind the irradiation of **1** is specifically produced by **5** rather than other low-lying isomers.

**Figure 4:**
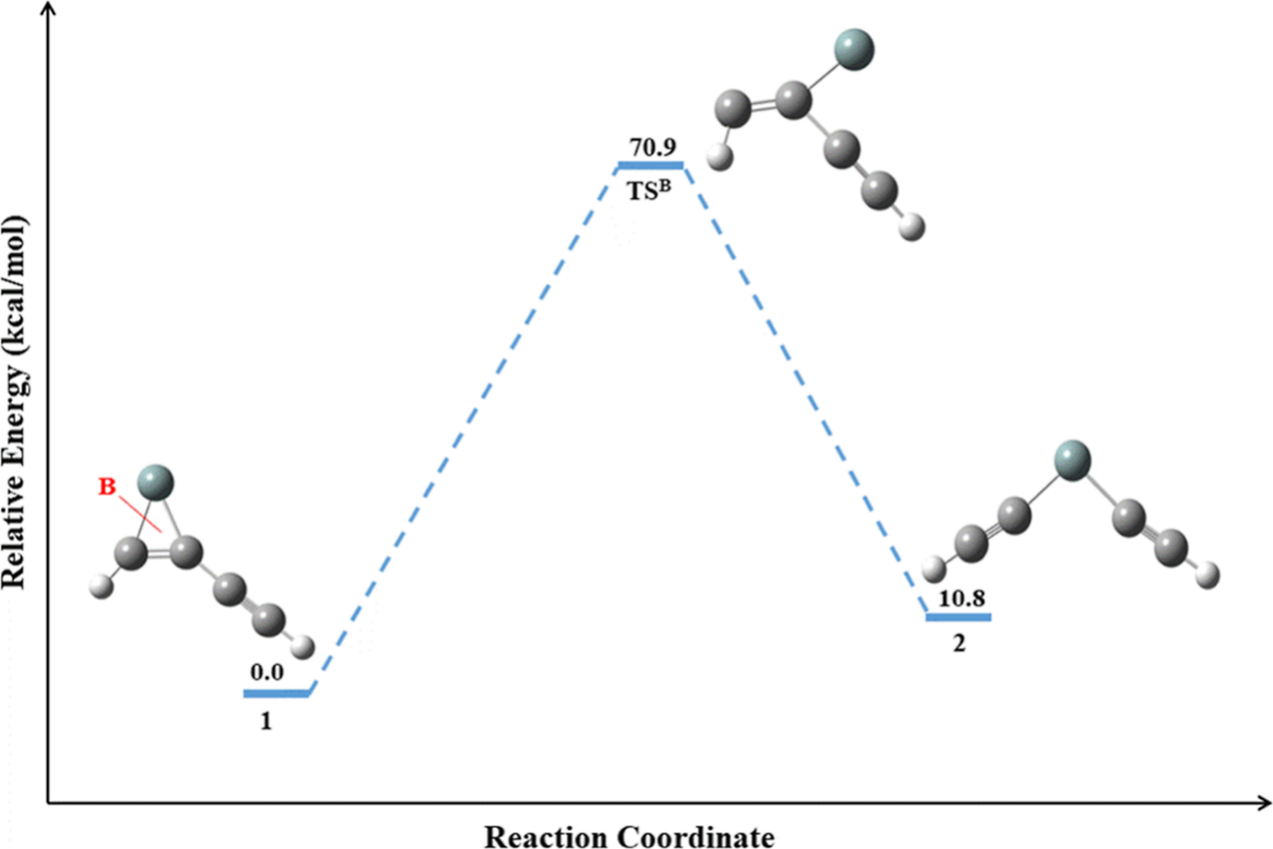
Reaction energy profile for the isomerization pathway of **1** to **2**. The ZPVE-corrected relative energy is in kcal/mol.

**Figure 5:**
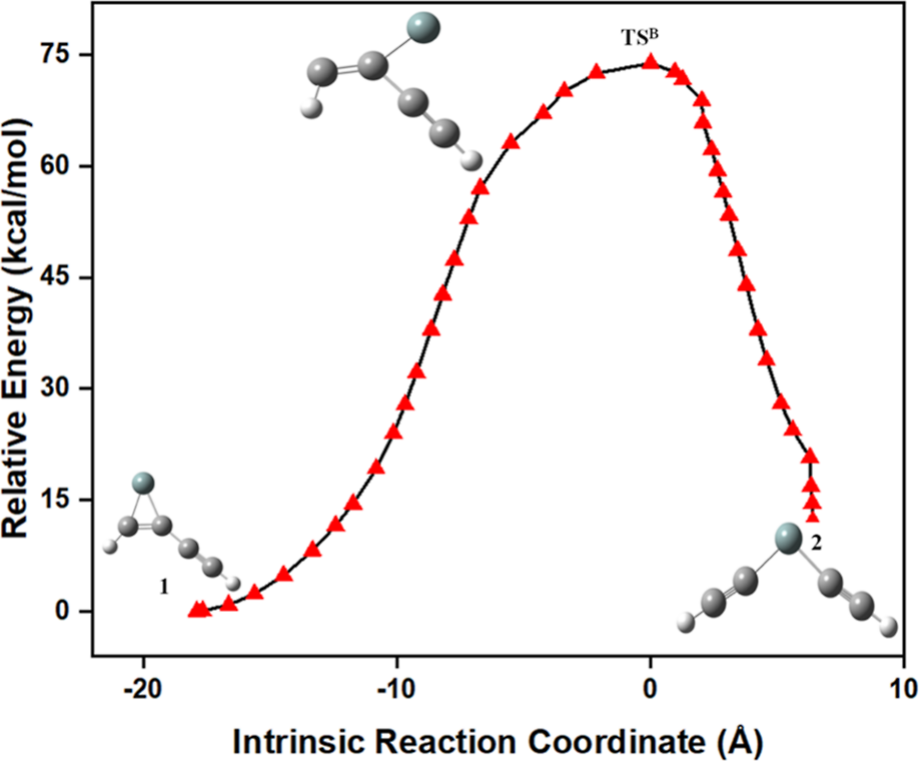
IRC for the isomerization pathway of **1** to **2**.

On the other hand, the dissociation pathway of type-B in **1** leads to the formation of **2**. The reaction energy profile for the isomerization pathway of **1** to **2** is shown in Figure 4. The identified transition state structure (**TS**^**B**^) for the isomerization of **1** to **2** follows an IRC pathway by connecting them through a minimum energy reaction path, as shown in Figure 5. The required activation energy for the isomerization of **2** from **1** is 70.9 kcal/mol. From this barrier height, one can conclude that **1** and **2** are the most kinetically stable isomers. Isomer **2** is an endothermic product when compared to **1**. The formation of **2** can happen by breaking the Si–C single bond in **1** through the dissociation pathway of type-B. The optimized geometrical parameters for the isomerization pathway of **1** to **2** and its corresponding transition state structure are listed in Table S3.

**Figure 6:**
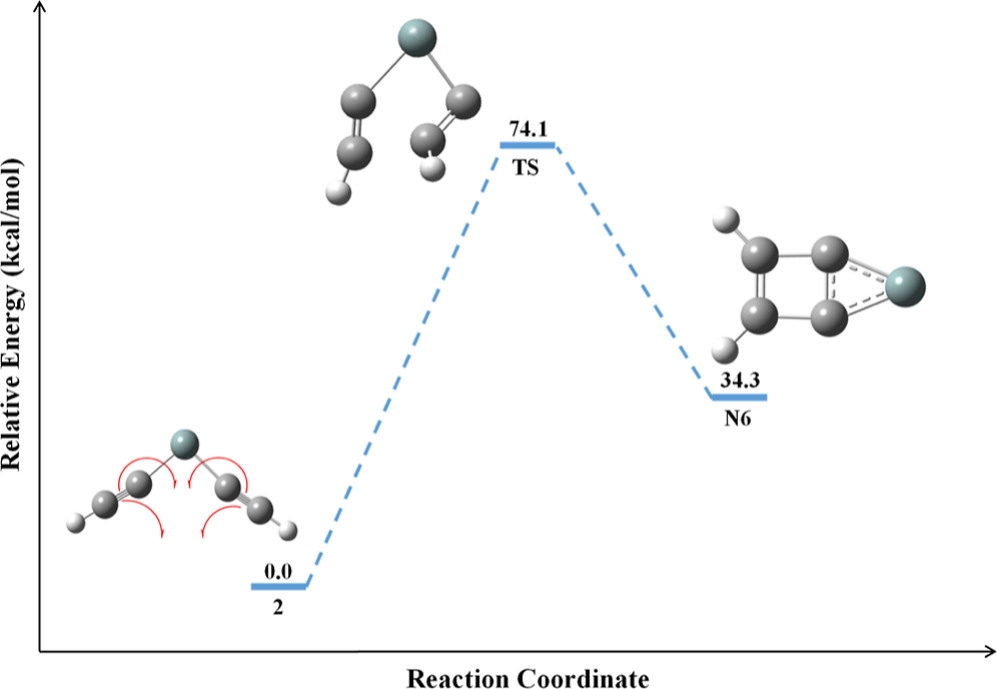
Schematic reaction energy profile for the formation of **N6**. The ZPVE-corrected relative energy is in kcal/mol.

**Figure 7:**
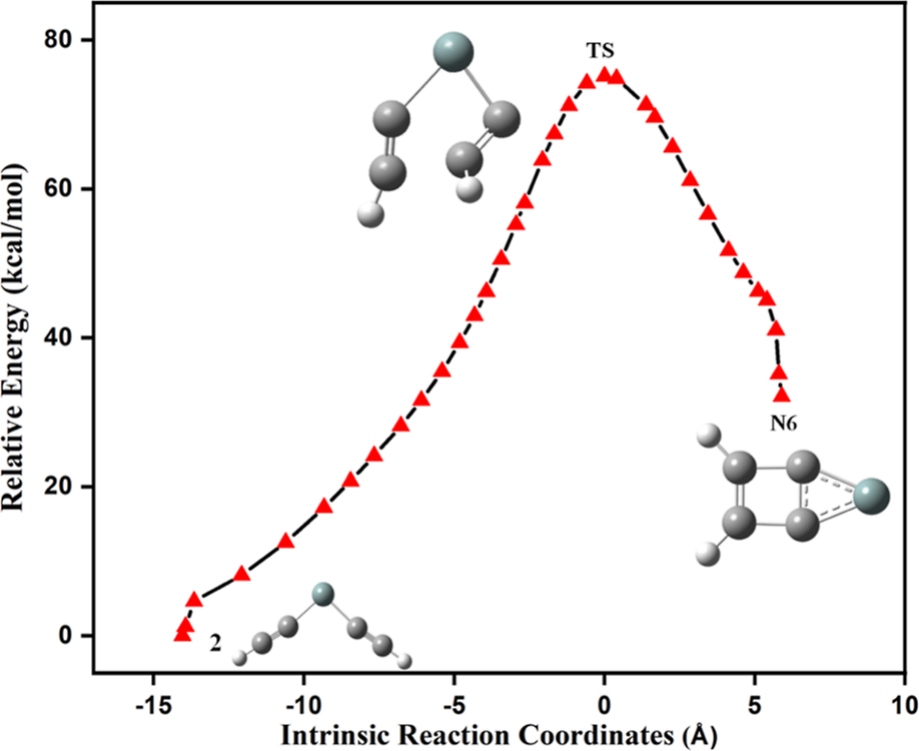
IRC for the isomerization pathway of **2** to **N6**.

The schematic reaction energy profile for the formation of **N6** is shown in Figure 6. The identified transition state structure for the formation of **N6** from **2** connects the minimum energy IRC path, as shown in Figure 7. The energy and geometry of the isomers in this reaction pathway are well connected to the IRC path. The predicted transition state structure for forming **N6** from **2** is a fused three-membered and four-membered ring with four carbon atoms formed through the radical mechanism. The required activation energy barrier for the reaction pathway is 74.1 kcal/mol, and the reaction is highly endothermic; thus, it dictates that **2** is a kinetically stable isomer. It is noted here that isomer **N6** is identified through present kinetic studies and is not reported elsewhere. The energy-minimized geometrical parameters for the isomerization pathway of **2** to **N6** and its corresponding transition state are provided in Table S4.

**Figure 8:**
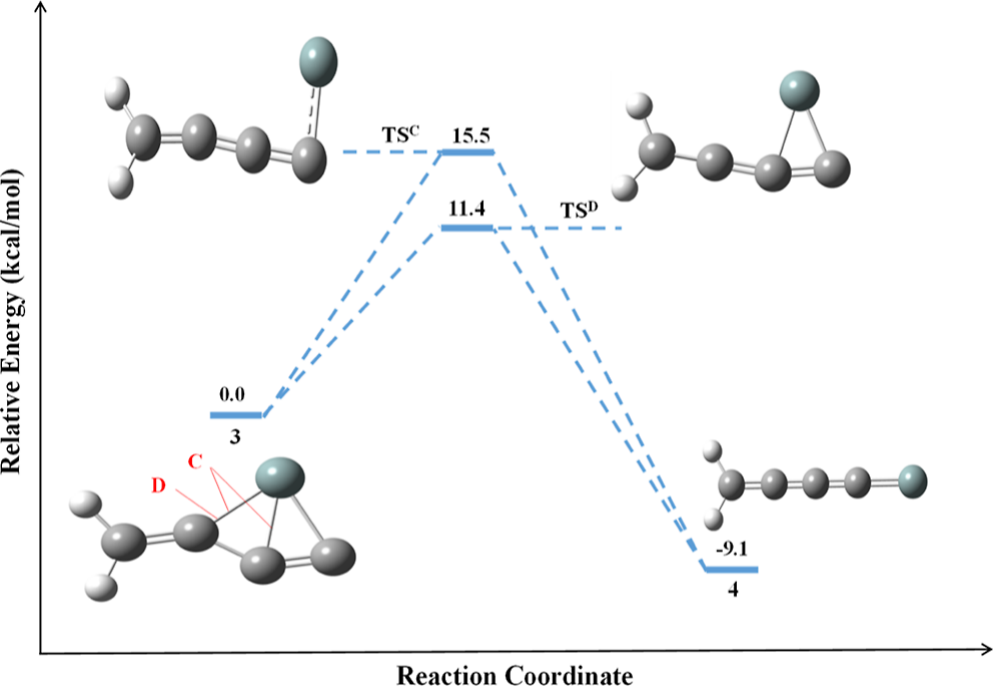
Schematic reaction energy profiles for the formation of **4** from **3**. The ZPVE-corrected relative energy is in kcal/mol.

**Figure 9:**
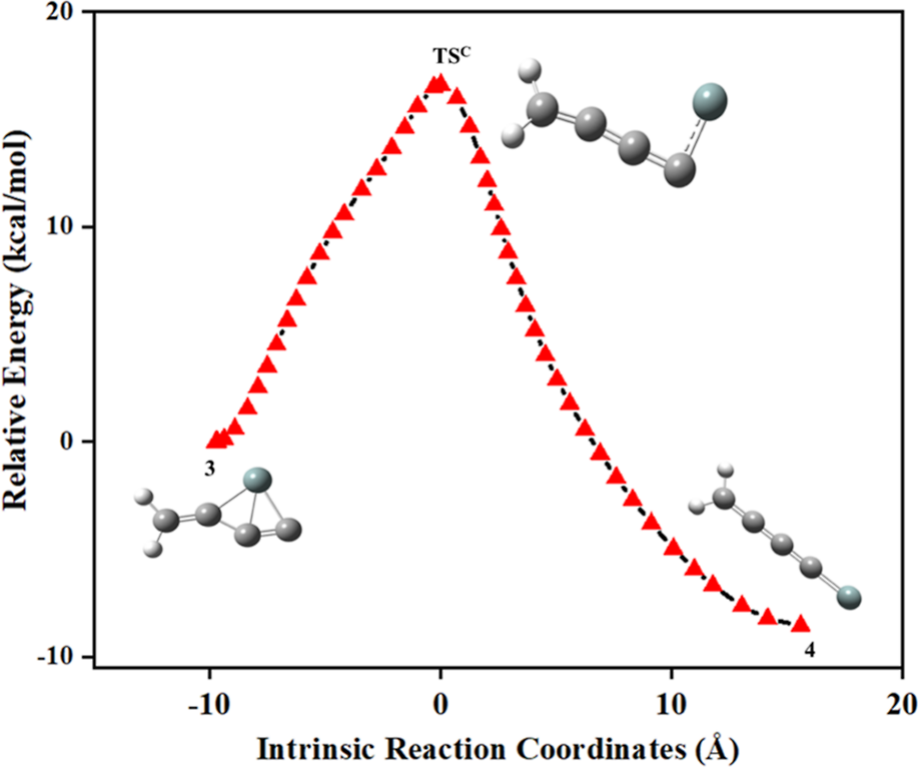
IRC for the isomerization pathway of **3** to **4**.

The isomerization pathways of type-C and type-D for the formation of **4** from **3** are shown in Figure 8. The dissociation of the Si–C bond in **3** leads to the second most thermodynamically stable isomer **4**, which is an exothermic product. The identified transition state structure (**TS**^**C**^) through the dissociation pathway of type-C for forming **4** is well connected with its minimum energy path, as shown in Figure 9. The energy-minimized geometrical parameters for the isomerization pathway of **3** to **4** with **TS**^**C**^ are provided in Table S5.

The activation energy required through this pathway for the formation of **4** from **3** is 15.5 kcal/mol. Furthermore, isomer **3** also connects to **4** through another transition state (**TS**^**D**^) structure. The calculated activation energy barrier for the conversion of **3** to **4** with **TS**^**D**^ is 11.4 kcal/mol. The energy-minimized geometrical parameters for the isomerization pathway of **3** to **4** with **TS**^**D**^ are provided in Table S6. Although the type-D pathway appears energetically favorable for the formation of **4** as an exothermic product, we leave this discussion with a caveat that our AIMD calculations on the other hand have indicated that isomer **3** is also a kinetically stable molecule awaiting experimental confirmation. The Cartesian coordinates of all of the low-energy isomers and their corresponding transition state structures are provided in Tables S7 and S8, respectively, obtained at the B3LYP/6-311++G(2d,2p) level of theory.

### 3.2 Rate Coefficient for the Isomerization Reaction

**Figure 10:**
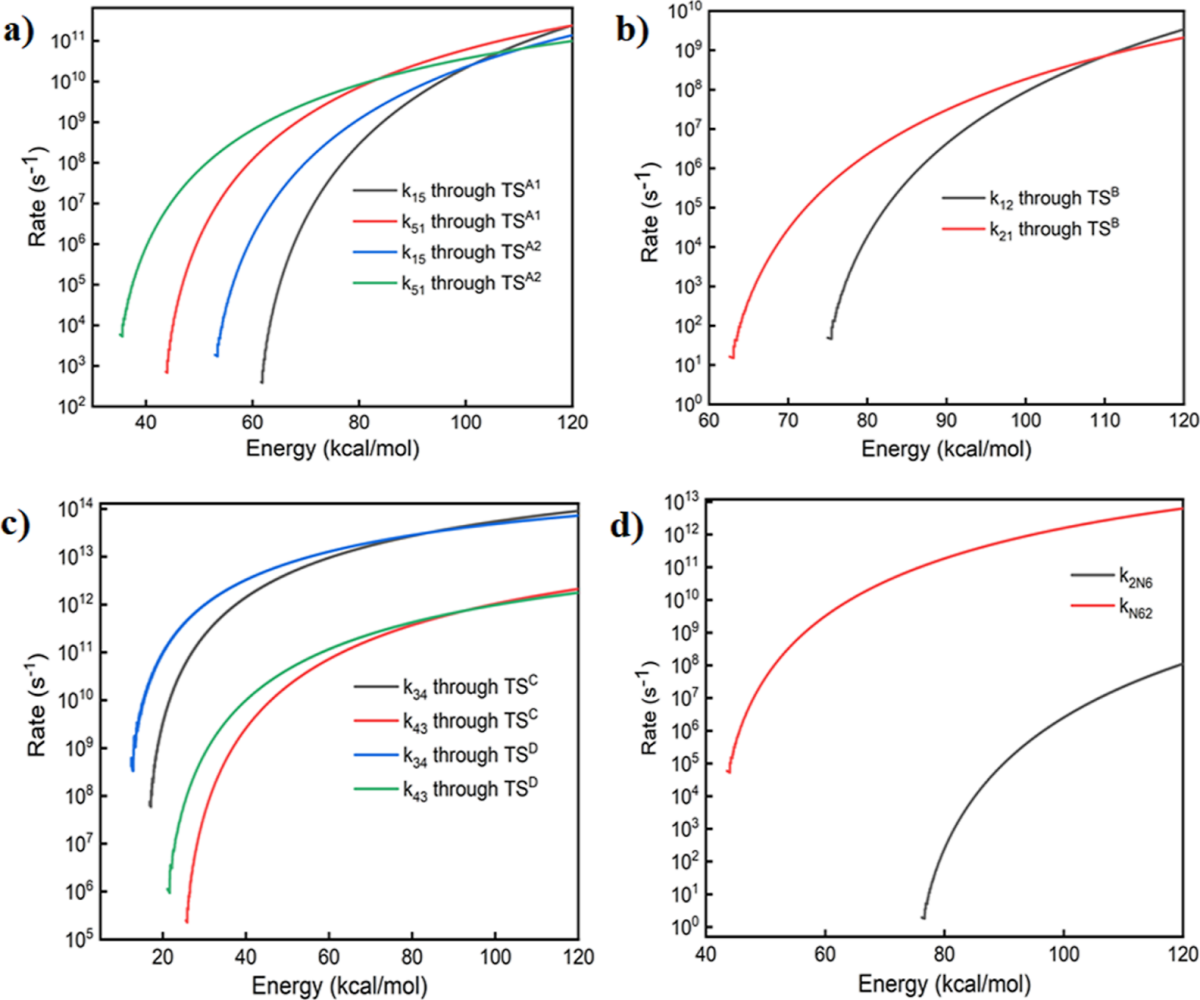
Rate coefficient for the forward and reverse isomerization reactions of (a) **1** and **5**, (b) **1** and **2**, (c) **3** and **4**, and(d) **2** and **N6**.

As mentioned previously, isomers **1**, **2**, **4**, and **5** are observed experimentally. However, isomers **3** and **N6** have not been identified in the laboratory until today. Therefore, the rate coefficients for investigated isomerization reactions are calculated to delineate the possibilities of finding these isomers in the experimental laboratory observations. As an energy function, the rate of isomerization reactions for **1** to **5**, **1** to **2**, and **3** to **4** are given in Figure 10. From the calculated rate coefficients, the equilibrium constant, the ratio of the forward to the reverse isomerization reactions, is calculated and provided as support information in Figure S1. The equilibrium constant is ∼1 order for the isomerization pathways of **1** and **5**, illustrating that the formation of both isomers is equally possible. In the case of **1** and **2**, the equilibrium constant is ∼10, indicating that the forward reaction (**1** to **2**) is 10 times faster than the backward reaction. Thus, the possible existence of isomer **1** is higher than **2**. Furthermore, the calculated rates for the isomerization of **2** to **N6** are provided. For these isomers, the rate of the isomerization between **N6** to **2** (reverse reaction) is several orders of magnitude faster than the rate of isomerization between **2** to **N6.**

Similarly, the isomerization rate from **3** to **4** with **TS**^**C**^ or **TS**^**D**^ is faster than the pathway of **4** to **3**, indicating that it reverts to **4**. This result reiterates the fact that, from a kinetic perspective, isomer **4** is kinetically more stable compared to **3**. Therefore, the possibilities of existing isomers **2** and **4** isomerizing to **N6** and **3** are significantly lower, which is easily understood from the equilibrium constant values. This may be one of the reasons why both **N6** and **3** have not been observed experimentally to date.

Overall, the isomerization pathways between the low-lying isomers of SiC_4_H_2_ are well documented here. The formation of **4** from **3** is kinetically and thermodynamically the most favorable pathway among the investigated isomers. Furthermore, this work shows that low-lying isomers of SiC_4_H_2_ can easily undergo potential rearrangements through Si–C bond dissociations. As per the experimentally observed isomerization in the Ar matrix at 10 K by Maier et al.,^41^ three-body interactions in the matrix can stabilize isomerized molecules when irradiated by 254 nm photons, thus stabilizing higher energy isomers. However, in ISM, three-body interactions are absent. Hence, this experimental method does not reproduce the actual astronomical environment. Even though the studied isomerization pathways may not be directly comparable with experimental protocols, they can help to predict the stability of these isomers. Therefore, the reverse reactions are important, which may hint at the stability of these isomers in the ISM.

### 3.3 Ab Initio Molecular Dynamics

**Figure 11:**
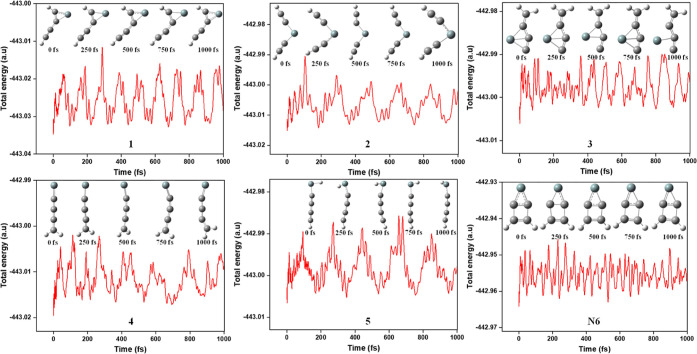
Total energy evolution of isomers **1**, **2**, **3**, **4**, **5**, and **N6** of SiC_4_H_2_ obtained from the AIMD simulation carried out at 10 K and 1 atm pressure at the UB3LYP/6-311++G(2d,2p) level.

Ab initio molecular dynamics simulations are carried out to demonstrate the stability of the lowest-lying isomers of SiC_4_H_2_ and also to determine whether these isomers would remain stable under the ISM conditions. The ADMP method,^52^ implemented in the Gaussian 16 program,^51^ was used to perform these calculations. These simulations were performed at 10 K and 1 atm pressure for 1000 fs. The time evolutions with total energies for the six isomers are depicted in Figure 11. In addition, snapshots at intervals of 250 fs are provided to analyze each isomer’s geometric changes within the simulation time scale. These data illustrate steady geometries across the whole period and a balanced oscillation of energies; thus, it indicates isomerization does not occur quickly, complimenting the high energy activation barriers of the potential energy surface profile of the isomerization process. As a result, it is also possible to deduce that these molecules are kinetically stable.

The formation of **4** from **3** follows an exothermic profile and is the most favorable pathway among the investigated isomers. Although this indirectly implies that isomer **3** is kinetically less stable compared to **4** through the proposed isomerization pathway, the molecular dynamics study of isomer **3** indicates that it is a kinetically stable molecule as such under cold conditions. At around 500 fs (see Figure 11), the Si–C bond breaks but then reforms again after a specific time (750 fs). Thus, one could conclude that **3** is also a kinetically stable molecule through AIMD simulation studies.

## 4 Conclusions

The low-lying isomers of SiC_4_H_2_ and their dissociation pathways with Si–C bond breaking in each isomer are theoretically investigated. Thermodynamically, all the isomers lie within ∼50 kcal/mol. The dissociation pathways of these isomers are predicted through transition state structure identification and are further confirmed by IRC calculations. These isomers’ kinetic and thermodynamic stabilities are well addressed through quantum chemical calculations. Isomers **1**, **2**, **4**, and **5** were reported in the laboratory, whereas **3** and **N6** remain elusive. Thermodynamically, isomers **3** and **5** are equally stable because they are not energetically well separated from each other. Also, the proposed isomerization pathway suggests that isomer **4** is kinetically more stable compared to isomer **3**. Nevertheless, the molecular dynamics data of isomer **3** indicate that it is also a kinetically stable molecule since the Si–C bond breaks but then reforms again after a specific time. Therefore, the question is whether the formation of **3** in the electrical gas chamber is possible by utilizing new precursor molecules. The calculated rate coefficients and equilibrium constant values elucidate the experimental difficulties in finding **N6** in the laboratory. This may be because **N6** will quickly revert to **2** according to the proposed isomerization pathways. Furthermore, the present theoretical work is expected to encourage and support experimental chemists in developing practical synthetic approaches and characterizing these putative isomers in terrestrial laboratory environments.

## Supporting Information Available

Optimized structural parameters of isomers and transition state structures obtained at the B3LYP/6-311++G (2d, 2p) level; Cartesian coordinates of the optimized geometries; total electronic energies, zero-point vibrational energies, free energies, and relative energies calculated at the B3LYP/6-311++G (2d, 2p) level; and rate coefficient for the isomerization reaction (PDF)
