# Supplementary material for: Theoretical Studies on the Isomerization Kinetics of Low-Lying Isomers of the SiC4H2 System
Source: J Phys Chem A. 2023 Dec 20;128(1):73–80. doi: 10.1021/acs.jpca.3c05658 (PMC10979431; doi:10.1021/acs.jpca.3c05658)
Supplement: Supplementary file 1 — jp3c05658_si_001.pdf [file jp3c05658_si_001.pdf]

Supporting Information

Theoretical Studies on the Isomerization Kinetics of Low-Lying Isomers of SiC_4H_2 System

Nisha Job[1], Vijayanand Chandrasekaran[1], Venkatesan S. Thimmakondu[2]* and Krishnan Thirumoorthy[1]*

[1] Department of Chemistry, School of Advanced Sciences, Vellore Institute of Technology, Vellore 632 014, Tamil Nadu, India

[2]Department of Chemistry and Biochemistry, San Diego State University, San Diego, CA 92182-1030, USA

vthimmakondusamy@sdsu.edu & kthirumoorthy@gmail.com

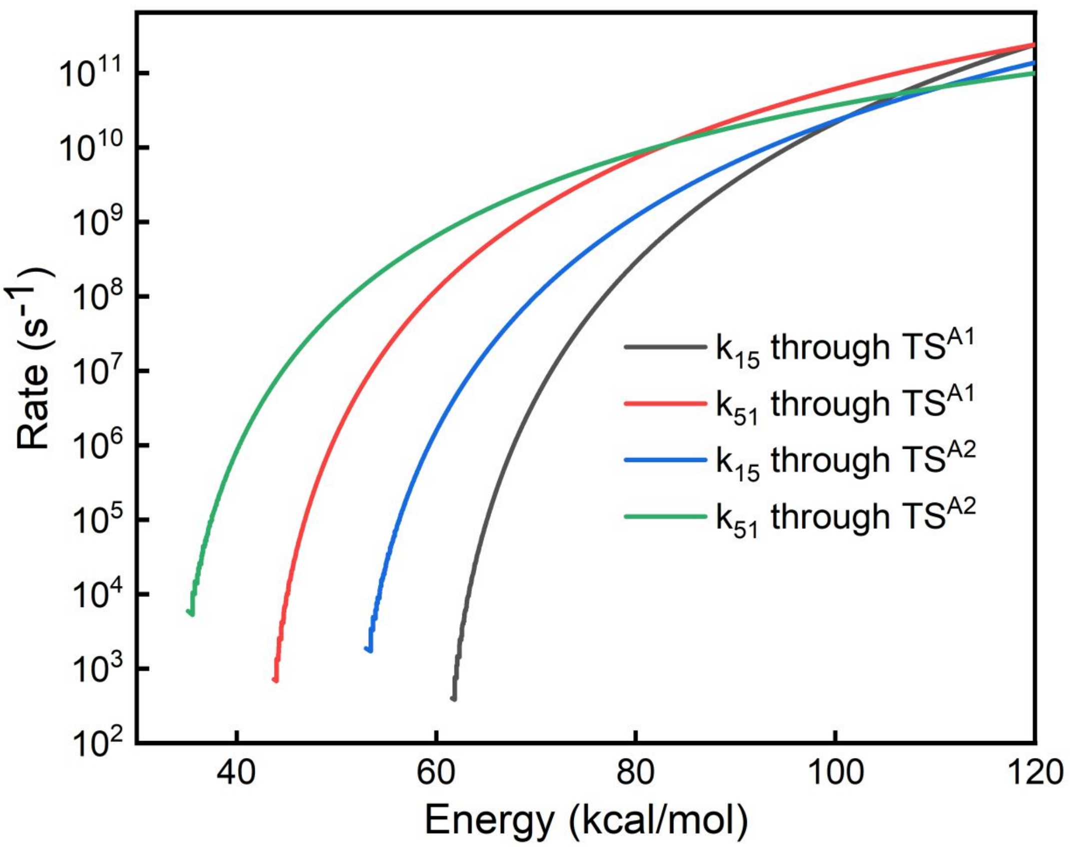

Figure S1. Rate co-efficient for the forward and reverse isomerization reactions of isomers **1** and **5**.

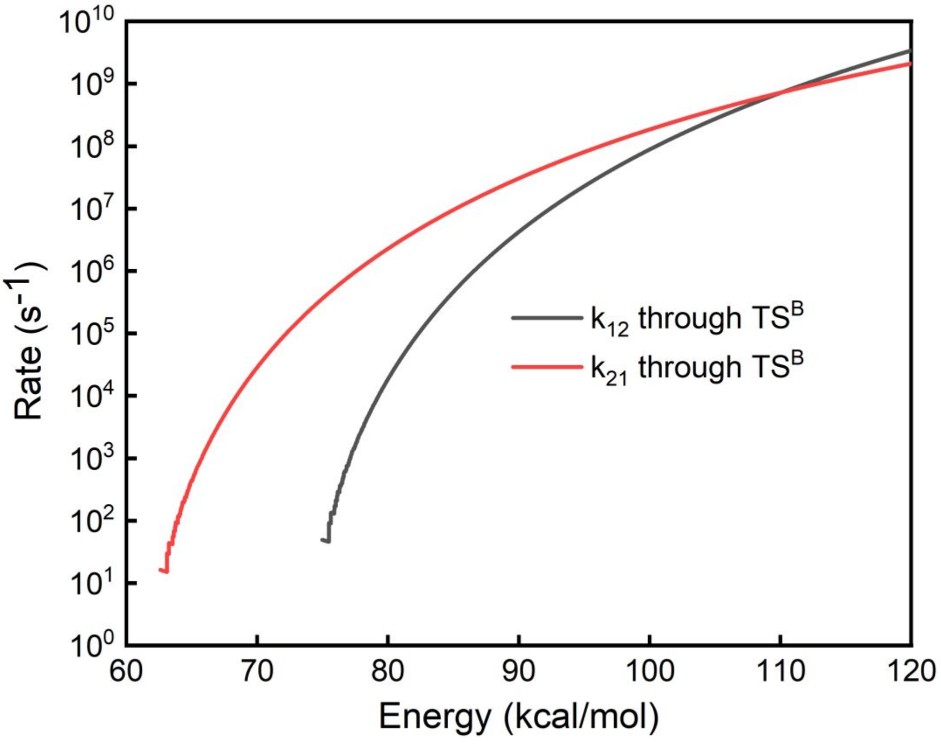

Figure S2. Rate co-efficient for the forward and reverse isomerization reactions of isomers **1** and **2**.

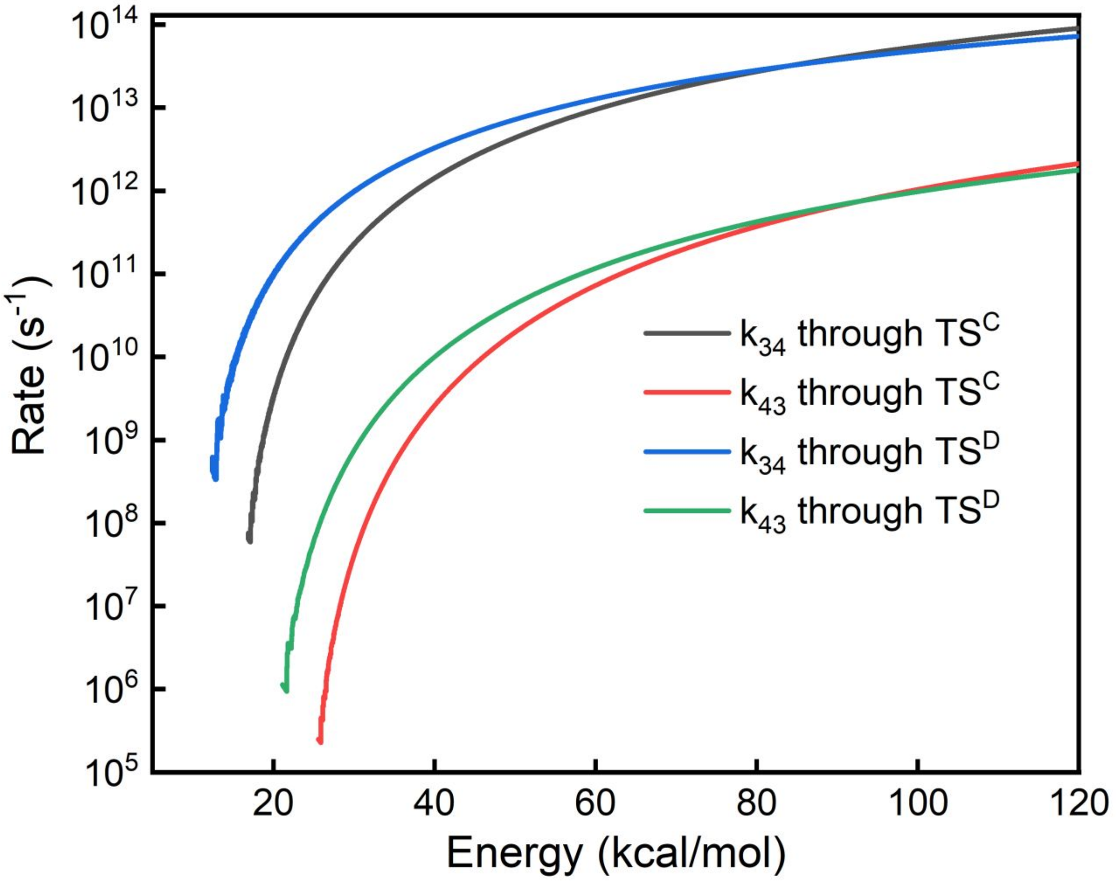

Figure S3. Rate co-efficient for the forward and reverse isomerization reactions of isomers **3** and **4**.

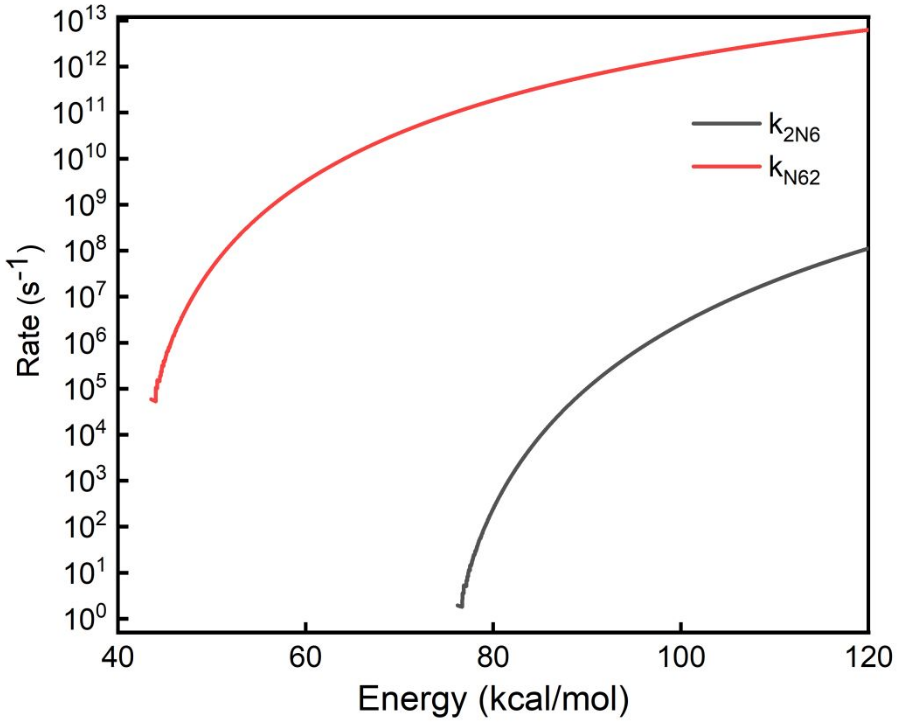

Figure S4. Rate co-efficient for the forward and reverse isomerization reactions of isomers **2** and **N6**.

Table S1: Optimized structural parameters (bond distances in Å and bond angles in Degree) for the isomerization of **1** to **5** and its corresponding transition state (TS^{A1}) as shown in Figure 2 at B3LYP/6-311++G (2d, 2p) level.

	**1**	**TS^{A1}**	**5**
$R(C_1C_2)$	1.3931	1.3620	1.3563
$R(C_1C_6)$	1.3498	1.3812	1.2253
$R(C_2C_3)$	1.2060	1.2223	1.2077
$R(C_3H_4)$	1.0607	1.0609	1.0613
$R(C_6H_7)$	1.0815	1.1161	-
$R(C_6Si_5)$	1.8198	1.7464	1.8289
$R(C_1Si_5)$	1.8489	-	-
$R(Si_5H_7)$	-	-	1.5208
$\theta(C_1C_2C_3)$	177.68	174.86	179.80
$\theta(C_2C_3H_4)$	179.65	174.69	179.90
$\theta(C_2C_1C_6)$	154.48	124.97	179.57
$\theta(C_1C_6Si_5)$	69.57	130.82	171.71
$\theta(H_7C_6C_1)$	132.98	120.72	-
$\theta(H_7Si_5C_6)$	-	-	93.78

Table S2: Optimized structural parameters (bond distances in Å and bond angles in Degree) for the isomerization of **1** to **5** and its corresponding transition state (TS^{A2}) as shown in Figure 2 at B3LYP/6-311++G (2d, 2p) level.

	**1**	**TS^{A2}**	**5**
$R(C_1C_2)$	1.3931	1.3679	1.3563
$R(C_1C_6)$	1.3498	1.3817	1.2253
$R(C_2C_3)$	1.2060	1.2496	1.2077
$R(C_3H_4)$	1.0607	1.0683	1.0613
$R(C_6H_7)$	1.0815	1.0848	-
$R(C_6Si_5)$	1.8198	1.8538	1.8289
$R(C_1Si_5)$	1.8489	2.3737	-
$R(Si_5H_7)$	-	-	1.5208
$R(C_1Si_5)$	177.68	-	2.2921
$\theta(C_1C_2C_3)$	179.65	154.29	179.80
$\theta(C_2C_3H_4)$	154.48	153.41	179.90
$\theta(C_2C_1C_6)$	69.57	108.98	179.57
$\theta(C_1C_6Si_5)$	132.98	93.23	171.71
$\theta(H_7C_6C_1)$	-	124.29	-
$\theta(H_7Si_5C_6)$	1.3931	-	93.78
$\theta(C_1Si_5C_6)$	1.3498	-	35.53
$\theta(C1Si_5C3)$	1.2060	-	66.29

Table S3: Optimized structural parameters (bond distances in Å and bond angles in Degree) for the isomerization of **1** to **2** and its corresponding transition state (TS^B) as shown in Figure 4 at B3LYP/6-311++G (2d, 2p) level.

	**1**	**TS^B**	**2**
$R(C_1C_2)$	1.3931	1.4960	-
$R(C_1C_6)$	1.3498	1.3656	1.2116
$R(C_2C_3)$	1.2060	1.2169	1.2116
$R(C_3H_4)$	1.0607	1.0625	1.0629
$R(C_6H_7)$	1.0815	1.0977	1.0629
$R(C_6Si_5)$	1.8198	-	-
$R(C_1Si_5)$	1.8489	1.7654	1.8468
$R(Si_5H_7)$	-	-	-
$R(C_2Si_5)$	-	-	1.8468
$\theta(C_1C_2C_3)$	177.68	170.45	-
$\theta(C_2C_3H_4)$	179.65	178.42	178.63
$\theta(C_2C_1C_6)$	154.48	128.36	-
$\theta(C_1C_6Si_5)$	69.57	-	-
$\theta(H_7C_6C_1)$	132.98	112.93	178.63
$\theta(C_2C_6Si_5)$	154.47	81.88	-
$\theta(C_6C_1Si_5)$	-	-	169.99
$\theta(C_6Si_5C_1)$	-	-	97.66
$\theta(C_3C_2Si_5)$	-	-	169.99

Table S4: Optimized structural parameters (bond distances in Å and bond angles in Degree) for the isomerization of **2** to **N6** and its corresponding transition state (TS) as shown in Figure 6 at B3LYP/6-311++G (2d, 2p) level.

	**2**	**TS**	**N6**
$R(C_1Si_2)$	1.8468	1.8364	1.8128
$R(Si_2C_3)$	1.8468	2.0229	1.8127
$R(C_3C_5)$	1.2116	1.2542	1.5411
$R(C_5H_7)$	1.0629	1.0672	1.0812
$R(C_1C_4)$	1.2116	1.3295	1.5416
$R(C_4H_6)$	1.0629	1.0847	1.0812
$R(C_5C_4)$	-	-	1.3341
$R(C_1C_3)$	-	-	1.4074
$\theta(C_1Si_2C_3)$	97.66	79.24	45.68
$\theta(Si_2C_3C_5)$	169.99	134.51	155.81
$\theta(C_3C_5H_7)$	178.63	163.03	132.86
$\theta(C_1C_4H_6)$	178.63	130.91	132.83
$\theta(Si_2C_1C_4)$	169.99	98.44	155.77
$\theta(C_3C_1C_4)$	-	-	88.62
$\theta(C_1C_4C_5)$	-	-	91.36
$\theta(C_4C_5C_3)$	-		91.37

Table S5: Optimized structural parameters (bond distances in Å and bond angles in Degree) for the isomerization of **3** to **4** and its corresponding transition state (TS^C) as shown in Figure 8 at B3LYP/6-311++G (2d, 2p) level.

	**3**	**TS^C**	**4**
$R(Si_1C_2)$	1.9701	1.7481	1.6998
$R(C_2C_3)$	1.2893	1.3245	1.2877
$R(C_3C_4)$	1.4022	1.2914	1.2737
$R(C_4C_5)$	1.3250	1.3145	1.3130
$R(C_5H_6)$	1.0823	1.0879	1.0857
$R(C_5H_7)$	1.0804	1.0857	1.0857
$R(Si_1C_3)$	1.9458	-	-
$R(Si_1C_4)$	1.9585	-	-
$\theta(Si_1C_2C_3)$	69.76	89.36	180.00
$\theta(C_2C_3C_4)$	141.24	177.65	180.00
$\theta(C_3C_4C_5)$	140.68	167.32	180.00
$\theta(C_4C_5H_6)$	121.87	121.77	121.74
$\theta(C_4C_5H_7)$	120.76	122.24	121.74
$\theta(H_6C_5H_7)$	117.38	115.99	116.52
$\theta(C_2Si_1C_3)$	38.44	-	-
$\theta(C_3Si_1C_4)$	42.09	-	-
$\theta(Si_1C_4C_5)$	150.87	-	-

Table S6: Optimized structural parameters (bond distances in Å and bond angles in Degree) for the isomerization of **3** to **4** and its corresponding transition state (TS^D) as shown in Figure 8 at B3LYP/6-311++G (2d, 2p) level.

	**4**	**TS^D**	**3**
$R(Si_1C_2)$	1.9701	1.8670	1.6998
$R(C_2C_3)$	1.2893	1.3205	1.2877
$R(C_3C_4)$	1.4022	1.3169	1.2737
$R(C_4C_5)$	1.3250	1.3119	1.3130
$R(C_5H_6)$	1.0823	1.0863	1.0857
$R(C_5H_7)$	1.0804	1.0819	1.0857
$R(Si_1C_3)$	1.9458	1.9062	-
$R(Si_1C_4)$	1.9585	-	-
$\theta(Si_1C_2C_3)$	69.76	71.01	180.00
$\theta(C_2C_3C_4)$	141.24	165.82	180.00
$\theta(C_3C_4C_5)$	140.68	179.99	180.00
$\theta(C_4C_5H_6)$	121.87	121.43	121.74
$\theta(C_4C_5H_7)$	120.76	122.00	121.74
$\theta(H_6C_5H_7)$	117.38	116.57	116.52
$\theta(C_2Si_1C_3)$	38.44	40.92	-
$\theta(C_3Si_1C_4)$	42.09	-	-
$\theta(Si_1C_4C_5)$	150.87	-	-

Table S7: Cartesian coordinates of 1 to N6 of SiC_4H_2 in Ångström at B3LYP/6-311++G (2d, 2p) level.

**1**				**2**			
C	0.00000	0.12572	0.00000	C	0.00000	1.39017	-0.17752
C	1.15219	0.90872	0.00000	Si	0.00000	0.00000	1.03823
C	2.17632	1.54559	0.00000	C	0.00000	-1.39017	-0.17752
H	3.07381	2.11132	0.00000	C	0.00000	2.42702	-0.80442
Si	-0.93223	-1.47096	0.00000	C	0.00000	-2.42702	-0.80442
C	-1.33808	0.30297	0.00000	H	0.00000	3.32315	-1.37596
H	-1.96513	1.18412	0.00000	H	0.00000	-3.32315	-1.37596
**3**				**4**			
C	0.00000	0.82699	0.00000	Si	0.00000	0.00000	2.48565
C	-0.10480	2.14784	0.00000	C	0.00000	0.00000	0.78584
Si	-0.81522	-0.95380	0.00000	C	0.00000	0.00000	-0.50181
C	0.97172	-0.18386	0.00000	C	0.00000	0.00000	-1.77547
C	1.08654	-1.47096	0.00000	C	0.00000	0.00000	-3.08852
H	0.76626	2.79017	0.00000	H	0.00000	0.92332	-3.65967
H	-1.07399	2.62531	0.00000	H	0.00000	-0.92332	-3.65967
**5**				**N6**			
H	1.01647	2.80990	0.00000	C	-0.23113	0.53390	0.28258
Si	-0.43386	2.35329	0.00000	Si	-1.45177	-0.33330	0.69312
C	0.00000	0.57668	0.00000	C	0.09286	-0.81787	0.28511
C	0.11706	-0.64302	0.00000	C	1.15106	0.50367	-0.07924
C	0.25716	-1.99208	0.00000	C	1.5258	-0.84890	0.14493
C	0.38597	-3.19283	0.00000	H	1.47176	1.50101	-0.29690
H	0.49641	-4.24836	0.00000	H	2.43202	-1.41779	0.14001

Table S8: Cartesian coordinates of transition state structures of SiC_4H_2 in Ångström at B3LYP/6-311++G (2d, 2p) level.

TS^{A1}				TS^{A2}			
C	0.44161	-0.15454	0.00282	C	0.4301434597	-0.9069389663	0.8903359523
C	1.79602	-0.09452	0.00416	C	1.6061770929	-0.2418586601	0.6402133493
C	3.01300	-0.14826	-0.00157	C	2.4118870127	0.4367130419	-0.0305664073
H	4.07391	-0.15464	-0.00891	H	3.3080666755	0.9862921793	-0.2157780978
Si	-2.23588	-0.2732	0.00017	Si	0.2108244276	0.8373804865	-0.6818626077
C	-0.62196	0.75332	-0.00773	C	-0.3634376662	-0.860597125	-0.2394237349
H	-0.54370	1.84343	0.02049	H	-1.3013774622	-1.3953192162	-0.3393486138
TS^{B}				TS			
C	-0.78984	0.02011	-0.02792	C	0.9351258973	1.0725142577	0.2080722578
C	0.63172	-0.58373	-0.03313	Si	2.0934219917	-0.3095572835	-0.1382459926
C	1.81033	-0.85655	0.06204	C	0.3222643932	-1.2876326296	-0.1494099668
H	2.84029	-1.09882	0.14144	C	-0.1933675101	0.4886829616	-0.1803070386
Si	0.16131	1.23392	0.02264	C	-0.7928283051	-1.0975867136	0.3922000498
C	-2.05492	-0.48547	0.11994	H	-0.9047403638	0.7525869122	-0.9550604354
H	-2.27409	-1.54025	0.28878	H	-1.7360618131	-1.2544699047	0.8661641757
TS^{C}				TS^{D}			
Si	2.51892	-0.18633	-0.22211	Si	1.17132	-0.70526	0.00008
C	1.28509	1.14825	-0.00889	C	1.23414	1.20336	-0.00009
C	0.06582	0.75162	-0.00884	C	0.01487	0.80672	-0.00004
C	-1.17264	-0.22191	-0.02886	C	-1.22358	-0.1668	-0.02005
C	-2.48742	0.02874	-0.02863	C	-2.53836	0.08384	-0.01983
H	-2.86271	1.04119	-0.02775	H	-2.91366	1.0963	-0.01895
H	-3.22287	-0.76618	-0.02913	H	-3.27381	-0.71107	-0.02033

Table S9: Computed ZPVE corrected energies of SiC_4H_2 isomers as shown in Figure **1**. The ground electronic states are calculated at the B3LYP/6-311++G (2d, 2p) level of theory.

**Isomer**	**E a.u**	**ZPVE a.u**	**E + ZPVE a.u**	**ΔE kcal mol^{-1}**	**ΔE + ZPVE kcal mol^{-1}**
**1**	-443.0436555	0.039323	-443.004333	0	0
**2**	-443.0241845	0.037096	-442.987089	12.22	10.82
**3**	-443.0149663	0.039876	-442.975091	18.00	18.35
**4**	-443.0286211	0.039006	-442.989615	9.43	9.24
**5**	-443.0156815	0.035014	-442.980668	17.55	14.85
**6**	-442.9729549	0.040521	-442.932434	44.36	45.12

Table S10: Computed Free energy corrected energies of SiC_4H_2 isomers as shown in Figure **1** and their ground electronic states calculated at the B3LYP/6-311++G (2d, 2p) level of theory.

Isomer	E<br>a.u	G<br>a.u	E + G<br>a.u	ΔE<br>kcal mol^{-1}	ΔE + G<br>kcal mol^{-1}
**1**	-443.0436555	0.011014	-443.032642	0	0
**2**	-443.0241845	0.007955	-443.016230	12.22	10.30
**3**	-443.0149663	0.012006	-443.002960	18.00	18.63
**4**	-443.0286211	0.011058	-443.017563	9.43	9.46
**5**	-443.0156815	0.006186	-443.009495	17.55	14.52
**6**	-442.9729549	0.013154	-442.959800	44.36	45.71

Table S11: Computed ZPVE corrected energies in the isomerization of SiC_4H_2 are shown in Figures **2**, **4**, **6**, and **8**. The ground electronic states are calculated at the B3LYP/6-311++G (2d, 2p) level of theory.

Structure		E<br>a.u	ZPVE<br>a.u	E + ZPVE<br>a.u	ΔE<br>kcal mol^{-1}	ΔE + ZPVE<br>kcal mol^{-1}
**Figure 2**	**1**	-443.0436555	0.039323	-443.004333	0	0
	**TS^{A1}**	-442.9469841	0.034776	-442.912208	60.66	57.81
	**5**	-443.0156815	0.035014	-442.980668	17.55	14.85
	**TS^{A2}**	-442.9604889	0.037330	-442.923159	52.19	50.94
**Figure 4**	**TS^{B}**	-442.9258495	0.034634	-442.891215	73.92	70.98
	**2**	-443.0241845	0.037096	-442.987089	12.22	10.82
**Figure 6**	**2**	-443.0241845	0.037096	-442.987089	0	0
	**TS**	-442.9044341	0.035495	-442.868939	75.14	74.14
	**N6**	-442.9729549	0.040521	-442.932434	32.15	34.30
**Figure 8**	**3**	-443.0149663	0.039876	-442.975091	0	0
	**TS^{C}**	-442.9884706	0.038051	-442.950420	16.63	15.48
	**4**	-443.0286211	0.039006	-442.989615	-8.57	-9.11
	**TS^{D}**	-442.9954541	0.038588	-442.956866	12.2	11.44

Table S12: Computed Free energy corrected energies in the isomerization of SiC_4H_2 are shown in Figures **2**, **4**, **6**, and **8**. The ground electronic states are calculated at the B3LYP/6-311++G (2d, 2p) level of theory.

**Structure**		**E** a.u	**G** a.u	**E+G** a.u	**ΔE** kcal mol^{-1}	**ΔE + G** kcal mol^{-1}
**Figure 2**	**1**	-443.0436555	0.011014	-443.032642	0	0
	**TS^{A1}**	-442.9469841	0.005675	-442.941309	60.66	57.31
	**5**	-443.0156815	0.006186	-443.009495	17.55	14.52
	**TS^{A2}**	-442.9604889	0.009775	-442.950714	52.19	51.41
**Figure 4**	**TS^{B}**	-442.9258495	0.005887	-442.919962	73.92	70.71
	**2**	-443.0241845	0.007955	-443.016230	12.22	10.30
**Figure 6**	**2**	-443.0241845	0.007955	-443.016230	0	0
	**TS**	-442.9044341	0.007312	-442.897122	75.14	74.74
	**N6**	-442.9729549	0.013154	-442.959800	32.15	35.41
**Figure 8**	**3**	-443.0149663	0.012006	-443.002960	0	0
	**TS^{C}**	-442.9884706	0.009285	-442.979185	16.63	14.92
	**4**	-443.0286211	0.011058	-443.017563	-8.57	-9.16
	**TS^{D}**	-442.9954541	0.010579	-442.984875	12.24	11.35
